# Maternal Vitamin B12 in Pregnancy and Placental Development

**DOI:** 10.1155/2024/3439995

**Published:** 2024-08-08

**Authors:** Amrita Arcot, Rachel E. Walker, Kelly Gallagher, Kevin C. Klatt, Alison D. Gernand

**Affiliations:** ^1^ Department of Nutritional Sciences The Pennsylvania State University, University Park, Pennsylvania, USA; ^2^ Ross and Carol Nese College of Nursing The Pennsylvania State University, University Park, Pennsylvania, USA; ^3^ Department of Nutritional Sciences and Toxicology University of California, Berkeley, California, USA

## Abstract

Vitamin B12, or cobalamin, is an essential nutrient required for diverse physiological functions secondary to its role as a critical cofactor for two mammalian enzymes, methionine synthase and methylmalonyl-CoA mutase. While essential throughout all life stages, several pathways that require vitamin B12, including hematopoiesis, myelination, and DNA/histone methylation, are particularly critical during pregnancy and fetal development. This narrative review aims to describe vitamin B12 in pregnancy, with emphasis on the placenta's role in ensuring adequate nutrition of the fetus and impacts of vitamin B12 deficiency on placental development and function. Our literature search included preclinical model systems and human cohorts and interventions. Our review identified evidence of B12 deficiency resulting in impaired placental development, greater placental inflammation, and modulation of placental docosahexaenoic acid concentration, collectively suggestive of vitamin B12 deficiency as a determinant of both maternal and fetal health outcomes. Heterogeneity in study design complicated generalization of findings. Future studies should consider selecting a B12 marker that is relatively stable across pregnancy, such as holotranscobalamin, while accounting for important confounders such as maternal folate.

## 1. Introduction

Vitamin B12, also known as cobalamin, is an essential B-vitamin required for the synthesis of two intracellular coenzymes, methylcobalamin and adenosylcobalamin [1]. Methylcobalamin serves as a cofactor for methionine synthase and is critical for the activation of S-adenosyl-methionine (SAMe), the universal methyl donor [2]. SAMe is needed for the methylation of various substrates, such as DNA, RNA, proteins, and the production of key products, such as phospholipids, neurotransmitters, and creatine [1]. Adenosylcobalamin serves as a cofactor for methylmalonyl-CoA mutase, catalyzing the conversion of methylmalonyl-CoA to succinyl-CoA and entry into the tricarboxylic acid cycle.

B12 deficiency can result in increased circulating homocysteine and methylmalonic acid [2]. In addition, B12 deficiency-induced disruptions to one-carbon fluxes in the folate cycle result in impaired nucleotide biosynthesis. These cellular defects collectively result in megaloblastic anemia and neurological complications in populations with inadequate B12 intake or impaired absorptive capacity [2–4]. Despite the high rates of hematopoiesis, nucleotide synthesis, and methylation reactions that are known to occur during pregnancy, the consequences of B12 deficiency in this physiological state remain less well described. Presently, there is limited information on maternal adaptations in B12 absorption and handling, the relative partitioning of B12 between the maternal and fetal compartments, and the key role of the placenta in coordinating this exchange. Furthermore, the impact of vitamin B12 on the processes of placentation and mature placental function has received limited attention, despite increasing recognition that such processes are sensitive to gestational micronutrient deficiency [5] and may be critical mediators of proximal adverse pregnancy outcomes as well as long-term neurocognitive and metabolic health [6–9]. Thus, the aim of this narrative review is to describe the published literature on B12 during pregnancy with a focus on B12 and placental development.

## 2. Main Text

### 2.1. B12 Sources

B12 is often recognized for its two unique features—its role in pernicious anemia [10] and its key availability in animal proteins [3]. Dietary vitamin B12 is typically found in animal products or bacteria, bound to proteins [1]. Of note, B12 is synthesized by bacteria and archaeon, not by animals or plants [11]. Ruminants will consume plants, which interact with B12-producing bacteria in their stomachs, resulting in B12 accumulation in animal tissue. Animals which eat a combination of plants and animals naturally increase their B12 consumption, which in turn will increase their B12 accumulation. Cumulatively, main sources of dietary B12 in humans are animal protein, eggs, milk, and other dairy foods [1]. Vegetarian and vegan diets inherently lack B12-rich sources, making those who follow such diets vulnerable to B12 deficiency [12]. Pregnant persons who follow vegetarian diets may be at increased risk of B12 deficiency, when compared to those following a standard Western diet [13–15].

### 2.2. Changes to Absorption in Pregnancy

Dietary vitamin B12 is found in animal products and bacteria, bound to proteins [1]. B12 digestion and absorption have five main stages: (1) release from proteins by hydrochloric acid and pepsin in the stomach [1], (2) binding to transcobalamin-I in the stomach, (3) release of B12 from transcobalamin-I by pancreatic enzymes with subsequent binding to intrinsic factor (IF) in the small intestine [16], (4) B12-IF uptake via receptor-mediated endocytosis into the enterocyte and subsequent recycling of IF in the small intestine [17], and (5) release of B12 into circulation, bound to transcobalamin-II (TC-II) [1]. A detailed description of B12 digestion and absorption has been previously published [18]. It is important to note that changes to B12 absorption during pregnancy may occur and need further investigation. The increase in progesterone during pregnancy can lead to delayed gastric emptying and increased intestinal transit time; presumably maximizing B12 absorption [19]. However, oral administration of drugs may have a slower time to maximum concentration, delaying their onset [20, 21]. Increased nausea and vomiting during early pregnancy can reduce nutrient absorption from dietary sources, and may further impede upon drug absorption. Importantly, our knowledge of digestion and absorption during pregnancy, especially in drug and supplemental trials, is limited out of concern for fetal health.

### 2.3. B12 Dietary Guidelines and Assessment

Nutrient requirements in pregnant populations are often derived from nonpregnant samples with adjustments for fetal growth, hematological needs, and the mobilization of maternal stores [22]. The B12 recommended dietary allowance (RDA) for pregnancy is 2.6 *μ*g, which is 0.2 *μ*g greater than the RDA for nonpregnant adults after accounting for fetal accumulation and increased maternal absorption [23]. Of the 47 studies used for dietary reference intake (DRI) recommendations by life stage, only 15% included pregnant or lactating [24]. Strikingly, of the nine studies used to set indicators for estimating B12, no study included pregnant or lactating persons. Dietary supplements are often recommended and consumed during pregnancy, as such we require more studies examining optimal dosing. The supplemental dosing of B12 far exceeds the established (RDA). According to the National Health and Nutrition Examination Survey (NHANES) data (1999–2014), a mean of 69.9% and 73.3% of pregnant persons self-reported dietary supplements containing B12 and folic acid, respectively [25]. The mean supplemental dosage of B12 was 16.8 *μ*g, among pregnant persons, which is approximately eight times higher than the RDA of 2.6 *μ*g/day [23]. Presently, B12 does not have a tolerable upper intake level since the maximum daily intake of B12, through fortified foods and supplements, and shows no evidence of toxicity and/or adverse events [23].

Recommended nutrient cut-offs in pregnancy are complicated by natural physiological changes, such as hormonal changes and plasma volume expansion [22]. Presently, there are limited data on optimal B12 biomarkers during pregnancy, resulting in unstandardized methods for assessing maternal B12 status. Total B12 is a common marker to evaluate vitamin status due to its cost and simplicity. However, B12 can decrease across pregnancy [26], which may be partly related to natural plasma volume expansion rather than a true tissue depletion [22]. The metabolically active fraction of B12, holo-TC, is considered a stable indicator of B12 status across pregnancy [26, 27], with one study setting an optimal cut-off at <62.2 (first trimester) and <67.5 pmol/L (second trimester; Table 1) [32]. Biospecimen collection did not occur in the third trimester, limiting these reference values to early- and mid-pregnancy, in exclusively European and South Asian pregnant persons. A separate follow-up [27] of a randomized control trial in Danish women [34] examined B12 biomarkers in 141 women. During gestation, cobalamin, total and holohaptocorrin, and methylmalonic acid declined across pregnancy, while holo-TC remained unchanged [27]. Examining holo-TC is expensive and is not widely accepted as a clinical indicator of B12 status. Furthermore, holo-TC is sensitive to recent intake (within hours), as such serum levels may increase but stores could remain low [28].

Methylmalonic acid (MMA) is also reported as a reliable indicator of B12 status; however, its analysis is both challenging and expensive. One study [35] examining pregnant persons reported a significant inverse correlation between vitamin B12 and MMA among pregnant persons, while another study [36] reported that increased MMA may not adequately represent suboptimal B12 levels during pregnancy. Elevated homocysteine can be used as an indicator for B12 deficiency; [2] however, there are issues regarding specificity. Homocysteine is a nonspecific marker of B12 as it is also influenced by methionine, folate, choline, and betaine intakes [2], and some studies find no correlation between B12 and homocysteine levels [35]. In light of these limitations, the assessment of B12 should be done with at least two markers [37].

### 2.4. B12 Deficiency in a Global Context

The prevalence of B12 deficiency globally is regarded as a public health concern; however, data on this are limited by surveys which vary from nationally representative to small and local contexts [38]. Furthermore, B12 markers and thresholds range across studies [39, 40]. Consequently, the extent of B12 deficiency globally is unclear. A systematic review examining global B12 deficiency during pregnancy reported a pooled rate of 25% [41]. The most striking was B12 deficiency in India which reported a pooled rate of 32%, 64%, and 60% for the first, second, and third trimesters, respectively. This may be in part related to dietary restrictions (e.g., vegetarian diets) from economic, cultural, and/or religious circumstances. Importantly, their review observed expansive heterogeneity across study design, B12 marker concentration cut-offs, use/reporting of B12 supplementation, and type of B12 marker [41]. Nationally representative data on B12 status during different life stages would better inform the global magnitude of B12 deficiency.

### 2.5. Placental Biology and Function

The placenta forms the interface between the pregnant person and the fetus, performing functions essential to fetal viability, growth, and development, including nutrient, gas, and waste product exchange, establishment of immune tolerance, and hormone production [42]. Upon fertilization, the inner cell mass of the blastocyst will develop into the embryoblast, and the outer cell mass will become the trophoblast [43, 44]. Trophoblasts differentiate into cytotrophoblasts and syncytiotrophoblasts which function to surround the placental chorionic villi and interact with maternal blood for nutrient and oxygen exchange [30, 42]. Maternal blood will enter the intervillous spaces of the placenta to bathe the chorionic villi. Maternal blood will provide gases and nutrients to the fetus through a single umbilical vein. Conversely, oxygen-depleted fetal blood and waste will flow through two umbilical arteries, back to the maternal body via the endometrial vein. Maternal health status can have implications on placental development and can impact fetal health. For example, maternal preeclampsia is a hypertensive disorder that occurs during pregnancy and can be exacerbated by multiple conditions such as renal disease, obesity, and pregestational diabetes [45]. Preeclampsia can result in inadequate spiral artery remodeling [46] in the placenta, and thus impede fetal growth (e.g., intrauterine growth restriction) due to impaired blood flow [47]. It is critical to understand the role maternal nutrition can have on placental development, transport, and function. The following sections aim to review the existing literature on maternal B12, placental function, and its clinical implications.

### 2.6. B12 and the Placenta

The connection between B12 and fetal or infant outcomes has been previously summarized [7]. Past evidence indicates a positive relationship between maternal B12 and cord blood B12, suggesting preferential transfer of B12 to the fetus. Schulze et al. (2020) conducted a substudy of a large randomized controlled trial in Bangladesh examining pregnant persons receiving either iron-folic acid (IFA) supplementation or antenatal multiple micronutrients (MM), which included 2.6 *μ*g of B12 [48]. They reported a strong positive correlation between maternal B12 and B12 in the cord blood plasma with a cord : maternal B12 blood ratio of 1.83 (95% confidence interval: 1.15, 2.91). This finding suggests active transfer of B12 from the mother to the fetus to meet fetal demands. To date, there is little information on the mechanisms of vitamin B12 transport across the placenta to the fetus.

**Figure 1:**
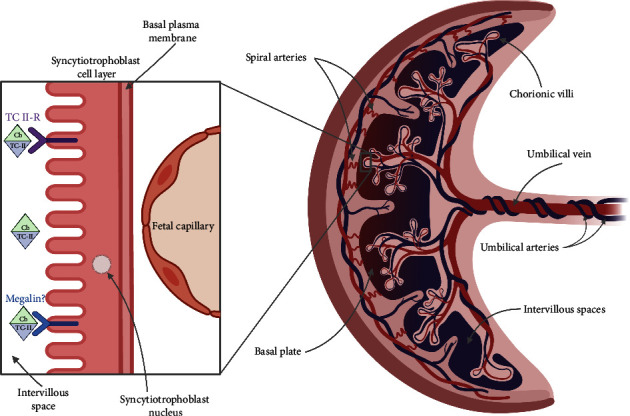
Theorized mechanisms for receptor-mediated endocytosis of cobalamin at the maternal-placental interface. Cb = cobalamin; TC-II = transcobalamin-II; TC II-R = transcobalamin II-receptor. Created with BioRender.com.

Investigators have theorized that TC receptors are expressed on the placenta for the binding and uptake of holo-TC for fetal circulation (Figure 1) [50, 49]. Receptor-mediated endocytosis via megalin may be another route for efficient placental B12 uptake, but additional research is needed [51]. The production of transcobalamin occurs in the placenta itself, during the early stages of pregnancy [53, 52]. A positive relationship between placental transcobalamin protein abundance and cord blood concentrations of vitamin B12 has been previously reported, suggesting increased placental transporter concentration to meet fetal demand [54]. Past evidence indicates time-sensitive expression of methionine synthase (MTR), metabolism of cobalamin-associated A (MMAA), and metabolism of cobalamin-associated B (MMAB) genes in mouse placentas [55]. MTR, MMAA, and MMAB genes were most abundant during early gestation, when the placenta is developing [56, 57]. Such findings may elucidate a relationship between maternal B12 status and its role in placental development to support fetal health.

Our review identified human, *in vitro*, and animal (rat) studies that examined placental outcomes and B12 with subsections examining *in vitro* and/or animal studies with folic acid, fatty acids, and homocysteine.  Table 2 details all studies identified.

### 2.7. Human Studies: B12 Status and Placenta Outcomes

We identified three human-subject studies which examine maternal B12 status and placental outcomes (Table 2) [54, 58, 59].

Mani et al., based in India, examined placental expression of angiogenic markers in 104 mother-infant dyads (18–33 years old) [59]. There was a significant negative association between B12 status and placental endoglin (ENG) and FMS-related receptor tyrosine kinase 1 (FLT) transcription. ENG, FLT, and vascular endothelial growth factor (VEGF) are linked to angiogenesis, and increased levels can contribute to the development of preeclampsia. Fetal consequences, if any, were not reported in this study, and B12 status was only collected in the first trimester. Layden et al., based in the United States, examined placental transcobalamin expression in 177 healthy adolescent pregnancies (13–18 years old) [54]. Investigators found no association between placental transcobalamin expression and maternal vitamin B12 concentration or dietary B12 intake [54]. Maternal blood samples were collected at midgestation and delivery. Notably, placental transcobalamin protein concentration was positively associated with cord blood vitamin B12, illustrating the importance of B12 to meet fetal demands.

Lastly, Bergen et al. was a prospective cohort in the Netherlands, which examined placental weight and vascular resistance, and its association with total homocysteine, in 5,805 pregnant women (15–46 years old) [58]. The investigators examined placental characteristics in relation to B12, homocysteine, and folate. Maternal biomarkers of this study were collected in early pregnancy (median: 13.2 weeks). The investigators reported no association between maternal B12 and placental outcomes (i.e., placental weight and placental vascular resistance) but they did not include quantitative results on B12. Women in the highest homocysteine quintile (≥8.3 *μ*mol/L) had a 30.1 gram reduction in placental weight, when compared to women in the reference homocysteine group (≤5.8 *μ*mol/L). Women in the highest quintile of homocysteine had an increased umbilical artery pulsatility index of 0.1, when compared to the reference, indicating an abnormal test of vascular resistance [65]. This association lost significance with adjustment for maternal and fetal characteristics. This suggests that B12 deficiency may be associated with lower placental weight and poor umbilical vascular function. However, elevated homocysteine is a nonspecific marker of one-carbon metabolism dysfunction.

Taken together, these studies found a negative association between maternal B12 status and placental angiogenic markers [59], and they found no association between maternal B12 status and transcobalamin expression [54] or vascular resistance [58]. Of note, cut-offs of B12 deficiency and time of B12 assessment were different across studies, highlighting the need for consistency in the measurement and examination of B12 in pregnancy.

### 2.8. *In Vitro* and Animal Studies: B12 and Folic Acid

Folate and folic acid are often examined with B12, due to their inextricable metabolic link in one-carbon metabolism [2]. High intake of folate and folic acid may impact placental outcomes, especially in the presence of B12 deficiency. Shah et al., who examined trophoblasts cell lines cultured in a folic acid and B12 deficient state, found that supraphysiological concentrations of folic acid (2000 ng/mL) reduced trophoblast viability and density, with improvements in both outcomes when these trophoblast cell lines were treated with B12 analogue (Table 2) [60]. They also noted a reduction in inflammatory markers, such as TNF*α*, when the trophoblast cells were treated with B12 analogues. Previous evidence supports a positive relationship between fetal growth and placental expression of miR-21 and miR-16 [66]. Shah et al. found that high folic acid consumption can reduce the placental expression of miR-21 and miR-16, with marked improvements in expression when treated with B12, especially with a combination of methylcobalamin and adenosylcobalamin (Table 2) [64]. All rats in this study were fed a B12 and folic acid deficient diet; however, female rats were dosed with folic acid (400 mcg) only one month prior to breeding. Dams were randomly assigned to their B12 treatment groups and both folic acid and B12 were administered by oral intubation.

These two studies illustrate the potential harm of high folic acid consumption in the absence of B12, which could impede placental development, increase inflammatory markers, and potentially increase the risk of fetal complications and poor fetal growth. Women of reproductive age who consume ≥400 *μ*g of folic acid daily has a mean erythrocyte folate concentration of 1,274 ng/mL [67]. This is nearly 1.5 times lower than the supraphysiological folic acid exposure in these studies. Importantly, the above *in vitro* studies examined trophoblast cell lines not erythrocytes, warranting caution in this comparison. Further investigation into physiologically realistic exposure and its impact on placental development is warranted.

### 2.9. Animal Studies: B12 and Placental Lipid Metabolism

One-carbon metabolism and vitamin B12 are integral to several lipid metabolism pathways, and these relationships are particularly important in the developing placenta. Long-chain polyunsaturated fatty acid (LC-PUFA) transport requires phosphatidyl choline (PC), which is dependent on methylation by phosphatidylethanolamine methyltransferase (PEMT) activity [68]. PEMT, in turn, is the predominant methyl acceptor from the one-carbon metabolism pathway, making LC-PUFA transport dependent on B12. PEMT transfers methyl groups to phosphatidyl ethanolamine (PE), converting it to phosphatidyl choline [69], the most important LC-PUFA-containing phospholipid for the placenta and developing fetus [70]. Changes in placental DHA are driven by circulating maternal DHA from tissue stores and diet. There are numerous studies showing that the placenta dramatically enriches the concentrations of DHA in the umbilical cord blood and fetal tissues by selective transport, a process called biomagnification [71–73]. During late pregnancy, mobilization of the fatty acids from adipose tissue is upregulated, resulting in a large influx of fatty acids to the placenta and fetus to be used for energy, growth, and development.

Early studies suggested that these increased nonesterified fatty acids from adipose were the source of DHA due to selective uptake by the fatty acid transport protein (FATP) family [71]. However, storage of DHA in adipose is relatively low, and most circulating DHA can be found in the phospholipid portion of lipoproteins. Therefore, it is likely that DHA uptake by the placenta comes primarily from lipolysis of the lipoproteins [74]. In fact, the predominant lipase present on the maternal face of the placenta is endothelial lipase, which has high phospholipolytic activity and a high affinity for both DHA and ArA [75]. DHA and ArA released from lipoproteins can then be available for uptake by the FATP proteins and are incorporated into PC in the trophoblasts by the action of PEMT. Therefore, B12 status may have implications for circulating and placental DHA concentrations.

The relationship between dietary B vitamins and omega-3 fatty acids in the placenta has been examined in several animal studies. Kulkarni et al. examined Wistar rats fed B12 deficient diets and reported significantly reduced placental DHA compared to the control group (Table 2) [61]. Placental DHA levels remained significantly lower among B12 deficient rodents, even in the presence of fatty acid supplementation. In addition, DHA supplementation ameliorated B12 deficiency-induced decreases in DNA methylation [61]. Wadhwani et al. reported significantly lower expression of placental Δ5 desaturase among B12 deficient Wistar rats, when compared to control rats, while placental Δ6 desaturase expression was unchanged. The Δ5 and Δ6 desaturase enzymes are critical steps in *de novo* LC-PUFA synthesis (Table 2) [62]. Together, these studies suggest that B12 concentrations may modulate placental DHA concentrations.

Although *de novo* synthesis of DHA may be important, placental synthesis of DHA is minimal compared to transplacental transport [71, 74]. DHA is transported across the placenta through the action of PEMT, which enriches PC with DHA through the conversion of PE-DHA to PC-DHA [76]. PC-DHA can then be transported to the fetus through packaging into lipoproteins in the placenta that are then released into the fetal circulation [77]. Past evidence reports increased plasma DHA concentrations among male rats fed diets enriched with folate, B12, and B6 [76]. Khot et al. reported significantly higher placental PEMT gene expression among B12 deficient pregnant rats receiving excess folic acid (8 mg/kg) or fatty acid supplementation, compared to controls [63]. These results suggest that PEMT expression could be a compensatory placental mechanism to ensure adequate provision of DHA to the fetus in the context of B12 deficiency (Table 2).

### 2.10. Animal Studies: B12 and Homocysteine

B12 deficiency precludes the conversion of homocysteine to methionine, which can result in hyperhomocysteinemia (≥15 *μ*mol/L) [78]. Elevated plasma homocysteine levels can result in placenta-mediated complications, such as small for gestational age (SGA) and pregnancy loss [79]. Kulkarni et al. reported no changes in plasma homocysteine levels when Wistar rats were fed diets deficient in B12 with either normal or excess folic acid, when compared to the control diet group (Table 2) [61]. In contrast, Shah et al. reported elevated homocysteine levels, in the presence of high folic acid and B12 deficiency [64]. Mechanisms may be present in the placenta to convert homocysteine to glutathione [63]. Increased expression of cystathionine beta-synthase (catalyzes the conversion of homocysteine to cystathionine) [2] in B12 deficient rats, consuming excess folic acid, has been previously reported [63]. This may indicate the presence of a compensatory mechanism to reduce homocysteine concentrations. Notably, cystathionine beta-synthase expression was not significantly different in other B12 deficient dietary groups, warranting further examination into the role of placental cystathionine beta-synthase during B12 deficiency, especially under conditions of excess folic acid intake.

## 3. Conclusion

Animal and *in vitro* studies have indicated that low B12 can reduce placental fatty acid uptake and synthesis, in part due to derangements in one-carbon metabolism. Furthermore, elevated folate and homocysteine levels, with B12 deficiency, could impede placental development, increase inflammation, and contribute to vascular resistance. However, the substantial methodological heterogeneity across studies does limit the ability to draw clear conclusions. Future research would benefit from examining B12 markers that are relatively stable throughout pregnancy—such as holo-TC in combination with other B12 markers (e.g., serum B12, MMA, and homocysteine). Furthermore, studies should examine maternal B12 when accounting for confounders such as maternal folate status, iron status, folic acid supplementation, DHA supplementation, and diet. A direct conclusion cannot be made between B12 and placental outcomes; however, this review highlights current gaps in our understanding of B12's role in placental development and function. There is a rich opportunity for examining B12 maternal status and placental development through untargeted analysis, via proteomic or metabolomic methods. Given B12's critical role in methylation, future studies should consider the intersection of maternal B12, fetal health, and epigenetics. Intervention studies examining B12 supplementation in a pregnancy cohort should consider placenta's sample collection to examine outcomes such as inflammation, vascular resistance, and fatty-acid concentration. Given the time and cost of human-participant analysis, ongoing *in vitro* work and animal model work are critical to examine placental development in B12 deficiency. From a public health perspective, ongoing research is needed on B12 cut-offs and its measurement during pregnancy. This will inform optimal B12 status during pregnancy and its mechanistic role in placental development, and as a result, fetal outcomes.

## Figures and Tables

**Table 1:** Methods of examining B12 status in nonpregnant and pregnant persons.

Marker	Reference value (B12 deficiency)	Method of measurement	Benefits	Drawbacks
B12	<150 pmol/L	ELISA	Simple measure Not greatly affected by recent intake [28]	Decreases may reflect plasma volume expansion [22, 28]

Holo-TC-II (active fraction)	<32 pmol/L (derived in nonpregnant persons) [31] ^*∗*^<62.2 (trimester one) and <67.5 pmol/L (trimester two) [32]	ELISA (MEIA) [33]	Relatively stable across pregnancy [27] May be best to test in late pregnancy [28]	Sensitive to recent intake—will increase even if stores are low Test cost [33] Not widely accepted as a clinical indicator Short half-life [29]

Methylmalonic acid	>0.27 *μ*mol/L	LC-MS/MS GC-MS ELISA	Greater specificity [2] Reflects liver stores [28]	Expensive Increases can be related to renal impairment or bacterial overgrowth [28] May not be reliable in pregnancy

Homocysteine	>15 *μ*mol/L	ELISA	Simple measure [2]	Low specificity

ELISA: enzyme-linked immunoassay; GC-MS: gas chromatography-mass spectrometry; LC-MS/MS: liquid chromatography-tandem mass spectrometry; MEIA: microparticle enzyme immunoassay; TC: transcobalamin. ^*∗*^Cross-sectional analysis of exclusively European and South Asian pregnant persons in Canada [32].

**Table 2:** Studies examining B12 and the placenta—human, *in vitro*, and animal.

Study, year (country)	Study type	B12 measurement and placental tissue collection	Main exposure	Control group	Main outcome	Main findings
*Human participants*						
Bergen et al., 2012 [58] (The Netherlands)	Prospective cohort	(i) Serum B12, plasma Hcy (ii) Collected in first and second trimesters (iii) Measured in quintiles (iv) Placental tissue not collected	N/A Folic acid supplement use examined but amount not reported	N/A	(i) Placental weight (ii) Placental vascular resistance (uterine and umbilical artery pulsatility index; third trimester) (iii) Birthweight (not reported in this table)	(i) Women in the highest Hcy quintile (≥8.3 *μ*mol/L) had a 30.1 gram reduction in placental weight, when compared to the reference group (Hcy ≤5.8 *μ*mol/L) (ii) Women with high Hcy (≥8.3 *μ*mol/L) concentration in late pregnancy had a higher umbilical artery pulsatility index of 0.11, compared to women in the reference (Hcy ≤5.8 *μ*mol/L), but this association lost significance with adjustment (iii) No associations were found with vitamin B12 and placental outcomes (data on maternal B12 and placental vascular resistance not reported)

Layden et al. 2016 [54] (United States)	Nested cohort	(i) Serum and cord blood B12 (ii) Collected at midgestation or at delivery (iii) Deficiency: <148 pmol/L (iv) Insufficiency cut-off <221 pmol/L (v) Placental tissue: four to five samples from the middle sections of different cotyledons	N/A Prenatal supplements containing iron (27 mg) and folic acid (400 *μ*g) as part of standard care	N/A	(i) Placental mRNA expression of TC (∆∆Ct) and abundance of TC protein (TC: *α*-actin) (ii) Presented as mean ± SD unless otherwise stated	(i) Maternal B12 concentration at mid-gestation and delivery were not associated with placental transcobalamin expression or protein abundance (ii) Serum B12 in the infant cord blood (662.1 ± 363.1) was significantly higher than maternal serum B12 at mid-gestation (348 ± 120.6) and at delivery (230.9 ± 95.2) (iii) Increased TC protein abundance was associated with greater infant cord blood vitamin B12 in univariate (*β* = 0.52 (SE = 0.16)) and multivariate (*β* = 0.48 (SE = 0.15)) analyses (iv) Male infant sex was positively associated in TC protein abundance in univariate (*β* = 0.35 (SE = 0.09)) and multivariate (*β* = 0.29 (SE = 0.09)) analyses
Mani et al. 2020 [59] (India)	Case-control	(i) Plasma B12 concentration (ii) Collected at first trimester (iii) Deficiency cut-off: <150 pmol/L (iv) Placental tissue: collected from the fetal side of the placenta	N/A Prenatal supplements included folic acid, iron, and calcium as part of standard care (amount not reported)	N/A	Placental ENG, FLT, and VEGF transcription levels	(i) Placental ENG and FLT transcript levels were negatively associated with maternal B12 status, with adjustment (ENG: *β* = −0.504; FLT: *β* = −0.583) (ii) Placental ENG and VEGF levels were negatively correlated with maternal B12 status for female births (ENG: *r* = −0.314, *p*=0.014; VEGF: *r* = −0.283, *p*=0.029) but not male (ENG: *r* = −0.107, *p*=0.489; VEGF: *r* = 0.059, *p*=0.702). No differences were found for SGA births, regardless of sex (iii) ENG, VEGF, and FLT expressions were not associated with placental weight or birth weight

*In vitro*						
Shah et al. 2016 [60] (India)	*In vitro*	(i) B12 measurement is not applicable (ii) Placental tissue: trophoblast-like cell lines, BeWo, and JEG3	(i) B12 treatment^*∗*^: 0.1, 1, 10, 100, and 1000 ng/mL (ii) Folic acid: normal physiological folic acid (NPFA) group: 20 ng/mL and supraphysiological folic acid (SPFA) group: 2000 ng/mL ^*∗*^100 ng B12/mL used to examine outcomes 2–4 in the main outcome column	(i) NPFA: 20 ng/mL (ii) SPFA: 2000 ng/mL	(i) Cell density (not reported in this table) (ii) Cell viability (not reported in this table) (iii) TNF*α*, EGFr, and *β*-hCG mRNA expression (iv) Homocysteine and MDA levels (lipid peroxidation) (v) Outcomes presented as mean ± SEM unless otherwise stated	(i) ^†^Placental TNF*α* mRNA expression was higher in the SPFA group, when compared to the NPFA group, with decreased expression when treated with B12 analogues (ii) ^†^Placental EGFr mRNA expression was lower in the SPFA group, when compared to the NPFA group, with increased expression when treated with B12 analogues (iii) Hcy levels in both cell lines were higher in the SPFA control (BeWo: 0.95 ± 0.16, JEG3: 0.76 ± 0.09), when compared to NPFA (BeWo: 0.47 ± 0.06, JEG3: 0.44 ± 0.07). Treatment with cobalamin (BeWo: 0.45 ± 0.07, JEG3: 0.41 ± 0.08) and methylcobalamin + adenosylcobalamin (BeWo: 0.42 ± 0.09, JEG3: 0.39 ± 0.09), in SPFA exposed cells, had lower Hcy concentrations, compared to the SPFA control (iv) BeWo cells, exposed to SPFA concentrations, had a reduction in *β*-hCG (9.93 ± 1.16 vs. 5.82 ± 0.43 (control)) and MDA (0.73 ± 0.07 vs. 1.12 ± 0.09 (control)) levels when treated with methylcobalamin + adenosylcobalamin. JEG3 cells, exposed to SPFA concentration, had a reduction in *β*-hCG (2.73 ± 0.58), compared to NPFA-exposed cells (5.14 ± 0.78) ^†^Results were depicted in figures, exact values not provided for inclusion in this table

*Animal*						
Kulkarni et al. 2011 [61] (India)	Wistar albino rats	(i) Plasma B12, tHcy (ii) Placental tissue collected, type not described	(i) Folic acid/-B12 (0.002 g folic acid/kg diet + 0.0 g B12/kg diet) (ii) Folic acid/-B12/+omegas^*∗*^ (0.002 g folic acid/kg diet + 0.0 g B12/kg diet) (iii) High folic acid/-B12 (0.008 g folic acid/kg diet + 0.0 g B12/kg diet) (iv) High folic acid/-B12/+omegas^*∗*^ (0.008 g folic acid/kg diet + 0.0 g B12/kg diet) ^*∗*^Fatty acid supplementation was in 1 : 1 ratio of omega-3 : omega-6	(i) Folic acid/+B12 (0.002 g folic acid/kg diet + 0.025 g B12/kg diet) (ii) High folic acid/+B12 (0.008 g folic acid/kg diet + 0.025 g B12/kg diet)	(i) Plasma folate, B12, Hcy, and fatty acid levels (ii) Placental fatty acid levels in different treatment groups (iii) Percent DNA global methylation (not reported in table) (iv) Outcomes presented as mean ± SD	(i) B12 levels were lower in the folic acid/-B12 (192.29 ± 28.53 pg/mL) and folic acid/-B12/+omegas (182 ± 17.52 pg/mL) compared to folic acid/B12 (287.63 ± 56.33 pg/mL) (ii) B12 levels in high folic acid/-B12 (188.25 ± 33.01 pg/mL) and high folic acid/-B12/+omegas (181 ± 25.63 pg/mL) were significantly lower, compared to folic acid/B12 and high folic acid/B12 (274 ± 70.58 pg/mL) (iii) Homocysteine levels were not different between groups (iv) Placental DHA was lower in the folic acid/-B12 (2.94 ± 0.99 pg/mL) and high folic acid/-B12 (2.76 ± 0.57 pg/mL), when compared to the control (folic acid/-B12: 3.73 ± 0.88 pg/mL)

Wadhwani et al. 2013 [62] (India)	Wistar albino rats	(i) B12 measurement was not described (ii) Placental tissue collected, type not described	(i) Folic acid/-B12 (2 mg folic acid/kg diet + 0.0 g B12/kg diet + 2.79 g ALA/100 g fatty acids) (ii) Folic acid/-B12/+omega-3^*∗*^ (2 mg folic acid/kg diet + 0.0 g B12/kg diet + 1.68 g ALA/100 g fatty acids) (iii) High folic acid/B12 (8 mg folic acid/kg diet + 0.025 g B12/kg diet + 2.70 g ALA/100 g fatty acids) (iv) High folic acid/-B12 (8 mg folic acid/kg diet + 0.0 g B12/kg diet + 2.91 g ALA/100 g fatty acids) (v) High folic acid/-B12/+omega-3^*∗∗*^ (8 mg folic acid/kg diet + 0.0 g B12/kg diet + 1.78 g ALA/100 g fatty acids) ^*∗*^5.64 g EPA/100 g fatty acids; 3.15 g DHA/100 g fatty acids ^*∗∗*^5.62 g EPA/100 g fatty acids; 3.13 g DHA/100 g fatty acids *Note*. Group names adapted in this table for clarity	(i) Folic acid/B12 (2 mg folic acid/kg diet + 0.025 g B12/kg diet + 2.29 g ALA/100 g fatty acids)	(i) Δ5 and Δ6 desaturase (ii) FATP1, FATP4, and FABP3 mRNA expression	(i) DHA and Δ5 desaturase mRNA levels were lower in the folic acid/-B12 and high folic acid/-B12 group, when compared to the control (ii) Δ6 desaturase mRNA levels reported no differences across groups, except for high folic acid/-B12/+omega-3 which had significantly higher Δ6 desaturase levels, when compared to all other diet groups. Similar findings were reported in Δ5 and Δ6 desaturase proteins (iii) FATP1 and FATP4 mRNA levels were significantly lower in rats fed B12 deficient diets compared to the control. FATP1 levels were not different to the control for rats fed B12 deficient diets with omega-3 supplementation (iv) FABP3 mRNA levels were nonsignificant across groups, except for folic acid/-B12/+omega-3 and high folic acid/-B12/+omega-3, which was significantly higher than high folic acid/-B12 and lower than folic acid/-B12/+omega-3, respectively *Note*. All described results are depicted in figures, exact values were not reported
Khot et al. 2014 [63] (India)	Wistar rats	(i) B12 measurement was not described (ii) Placental tissue collected, type not described	(i) Folic acid/-B12 (0.002 g folic acid/kg diet + 0.0 g B12/kg diet) (ii) Folic acid/-B12/+omega-3 (2 mg folic acid/kg diet + 0.0 g B12/kg diet + 120 mg DHA + 180 mg EPA) (iii) High folic acid/+B12 (0.008 g folic acid/kg diet) + 0.025 g B12/kg diet) (iv) High folic acid/-B12 (0.008 g folic acid/kg diet) + 0.0 g B12/kg diet) (v) High folic acid/-B12/+omega-3 (0.008 g folic acid/kg diet + 0.0 g B12/kg diet + 120 mg DHA + 180 mg EPA)	(i) Folic acid/+B12 (0.002 g folic acid/kg diet) + 0.025 g B12/kg diet)	(i) Placental mRNA levels of MTHFR, MTR, MAT2a, CBS, and PEMT (ii) Placental PC : PE ratio	(i) ^*∗*^B12 deficient murine had decreased expression of MTHFR and MTR, when compared to the control (ii) ^*∗*^Increased MAT2A, PEMT, and CBS gene expression if consuming high folic acid/-B12, when compared to control and high folic acid/+B12. No differences in expression were found between folic acid/-B12 and the control (iii) The PC : PE ratio was significantly higher in the high folic acid/-B12 group (1.81 ± 0.81), when compared to both the control (1.06 ± 0.53) and folic acid/-B12 (0.72 ± 0.35). The ratio among high folic acid/-B12/+omega-3 (0.83 ± 0.24) was lower than the high folic acid/-B12 group *Note*. Described results are depicted in figures, exact values not provided for inclusion in this table

Shah et al. 2017 [64] (India)	Wistar rats	(i) Plasma B12 and Hcy (i) Placental tissue collected, type not described	(i) Folic acid/methylcobalamin (ii) Folic acid/cobalamin (iii) Folic acid/hydoxycobalamin (iv) Folic acid/adenosylcobalamin (v) Folic acid/methylcobalamin + adenosylcobalamin^*∗*^ (vi) High folic acid/methylcobalamin (vii) High folic acid/cobalamin (viii) High folic acid/hydoxycobalamin (ix) High folic acid/adenosylcobalamin (x) High folic acid/methylcobalamin + adenosylcobalamin^*∗*^ Folic acid: 400 *μ*g High folic acid: 5 mg All B12 treatment analogues: 2.6 *μ*g total^*∗*^ ^*∗*^MeCbl (1.3 *μ*g) + AdCbl (1.3 *μ*g) = 2.6 *μ*g	(i) Folic acid (400 *μ*g) (ii) High folic acid (5 mg)	(i) Placental miR-16 and miRNA-21 expression (ii) Pup birthweight (iii) Placental weight (not reported in this table)	(i) miR-16 and miR-21 placental expression were lower in the high folic acid control diet with improvements when treated with B12, especially MeCbl + AdCbl (ii) Pup birthweight was lower among pregnant rats consuming HFol diets, with improvements in birth weight when treated with B12 analogues *Note*. Described results are depicted in figures, exact values not provided for inclusion in this table

AdCbl: adenosylcobalamin; ALA: alpha-linolenic acid; *β*-HCG: beta-human chorionic gonadotropin; Cbl: cobalamin; CBS: cystathionine *β*-synthase; DHA: docosahexaenoic acid; EGFr: epidermal growth factor receptor; ENG: endoglin; FA: folic acid; FATP: fatty acid transport protein; FABP3: fatty acid binding protein 3; FLT: FMS-related receptor tyrosine kinase 1; Hcy: homocysteine; MAT2a: methionine adenosyltransferase; MeCbl: methylcobalamin; MeCbl + AdCbl: methylcobalamin and adenosylcobalamin; MTHFR: methylene tetrahydrofolate reductase; MTR: methionine synthase; PC: phosphatidylcholine-DHA; PE: phosphatidylethanolamine-DHA; PEMT: phosphatidylethanolamine methyl transferase; SD: standard deviation; SEM: standard error of the mean; SGA: small for gestational age; TC: transcobalamin; tHcy: total homocysteine; TNF*α*: tumor necrosis factor alpha; VEGF: vascular endothelial growth factor.

## Data Availability

Data sharing was not applicable for this article as no datasets were generated or analyzed during the current study.
